# Electrical stimulation and biofeedback combined with pelvic floor muscle training for pelvic floor dysfunction in postpartum women: a systematic review and meta-analysis

**DOI:** 10.3389/fphys.2026.1832015

**Published:** 2026-09-07

**Authors:** Xiaoqi Huang, Yan Hu, Fujin Hu, Changhao Li, Xiaoli Huang, Yingwei Ai

**Affiliations:** 1 Institute of Artificial Intelligence in Sports, Capital University of Physical Education Sports, Beijing, China; 2 Army Health Service Training Base, Army Medical University, Chongqing, China; 3 Department of Obstetrics and Gynecology, People’s Hospital of Wenshan Prefecture, Wenshan, China; 4 Radiology Department, Yunnan Cancer Hospital, Kunming, China; 5 Radiology Department, Wenshan City Maternal and Child Health Care Hospital, Wenshan, China; 6 Guangzhou College of Commerce, Guangzhou, China

**Keywords:** biofeedback, electrical stimulation, meta-analysis, pelvic floor dysfunction, pelvic floor muscle training, postpartum women

## Abstract

**Background:**

Postpartum pelvic floor dysfunction (PFD) manifests as decreased pelvic floor muscle strength (PFMS), stress urinary incontinence (SUI), pelvic organ prolapse (POP), and impaired sexual function. Pelvic floor muscle training (PFMT) is the first-line therapy but often limited by poor muscle perception and adherence. Electrical stimulation (ES) and biofeedback (BF) may enhance outcomes, yet their combined effect with PFMT has not been systematically quantified.

**Methods:**

Following PRISMA 2020 guidelines, PubMed, Embase, Web of Science, Cochrane Library, CNKI, WanFang, VIP, and SinoMed were searched to July 9, 2026. Randomized controlled trials (RCTs) comparing ES+BF+PFMT with PFMT alone were included. The population was restricted to women within 12 months postpartum. Outcomes were PFMS, SUI incidence, POP severity, and sexual function. Pooled mean differences (MD) or risk ratios (RR) with 95% confidence intervals (CI) were calculated. Subgroup and sensitivity analyses were performed, publication bias was assessed for outcomes with at least 10 studies, and the certainty of evidence was evaluated using GRADE.

**Results:**

Twenty-nine RCTs were included. ES+BF+PFMT significantly improved PFMS (MD = 0.85, 95% CI: 0.69–1.01), reduced SUI incidence (RR = 0.36, 95% CI: 0.26–0.49), lowered POP-Q stage (MD=−0.29, 95% CI: −0.39 to −0.20), and enhanced sexual function (MD = 4.38, 95% CI: 3.41–5.34). Exploratory subgroup analyses suggested that Kegel training duration may contribute to PFMS heterogeneity. GRADE assessment indicated moderate certainty for SUI incidence, low certainty for PFMS and sexual function, and very low certainty for POP severity.

**Conclusions:**

Compared with PFMT alone, ES+BF+PFMT may provide additional benefits for postpartum PFD rehabilitation within the first year, with the most consistent evidence observed for reducing SUI incidence. Potential benefits were also observed for PFMS and sexual function, whereas the POP-Q findings should be interpreted with particular caution because the certainty of evidence was very low. The generalizability of these findings is limited because all included studies were conducted in China.

**Systematic Review Registration:**

https://www.crd.york.ac.uk/PROSPERO/view/CRD420251134152, identifier CRD420251134152.

## Brief summary

This meta-analysis suggests that postpartum women receiving pelvic floor muscle training combined with electrical stimulation and biofeedback may achieve better outcomes compared with training alone.

## 1 Introduction

Pelvic floor dysfunction (PFD) is a clinical syndrome characterized by impaired support capacity and structural damage of the pelvic floor muscles, ligaments, and fascia. It primarily manifests as stress urinary incontinence (SUI), pelvic organ prolapse (POP), sexual dysfunction, and pelvic pain (Huang et al., 2024). Pregnancy and childbirth are strongly associated with the onset of PFD and represent major contributing factors (Chen et al., 2023; Wu et al., 2023). During pregnancy, changes in body weight and hormone levels play pivotal roles. To counterbalance the anterior shift of the body caused by abdominal weight gain and breast enlargement, women often develop compensatory maladaptive postures such as lumbar hyperlordosis and kyphosis. Prolonged maintenance of these abnormal postures may lead to skeletal displacement, persistent tension of paraspinal and abdominal muscles, and restricted spinal flexion–extension and lateral movement. In addition, elevated levels of progesterone and relaxin during pregnancy disrupt the collagen fiber structure of the pelvic floor, reducing muscle support capacity, loosening ligaments, and decreasing the stability of the pubic symphysis and sacroiliac joints, thereby further increasing the burden on pelvic floor muscles (Wang et al., 2023). During childbirth, pelvic structures that support pelvic organs (muscles, ligaments, and fascia) undergo excessive stretching, which may result in neuromuscular injury and connective tissue weakness, subsequently leading to PFD (Wallace et al., 2019).

Epidemiological studies have shown that the incidence of SUI within 12 months postpartum ranges from 30% to 47% (Mantilla Toloza et al., 2024); the prevalence of POP varies between 2% and 50% (Basnet, 2021); and the prevalence of sexual dysfunction is approximately 29.7% (Radzimińska et al., 2018). These dysfunctions substantially impair the physical and psychological health of postpartum women and diminish their quality of life, constituting a major public health challenge. Therefore, identifying effective rehabilitation strategies for postpartum PFD has become a key focus of current medical research.

At present, interventions for postpartum PFD mainly include conservative therapy and surgical treatment. Conservative therapy is generally prioritized as the initial approach for postpartum pelvic floor rehabilitation and includes a spectrum of strategies, such as PFMT, lifestyle and behavioral interventions, physical therapy, biofeedback, and electrical stimulation (Poenaru et al., 2026). Among these conservative strategies, pelvic floor muscle training (PFMT), such as Kegel exercises, is widely recommended as the first-line treatment due to its noninvasiveness, safety, and cost-effectiveness (Huang et al., 2024). PFMT enhances pelvic floor muscle function through regular contraction–relaxation exercises (Villani et al., 2024). However, previous studies have indicated that most women with PFD exhibit poor adherence to PFMT and often fail to accurately perceive and control pelvic floor contractions, thereby limiting treatment effectiveness (Li et al., 2025b). To optimize outcomes, various physical therapies are frequently employed as adjuncts to PFMT, including electrical stimulation (direct activation of weak muscles or nerve fibers through current-induced passive contraction) (Chen et al., 2024) and biofeedback (assisting patients in perceiving and performing correct pelvic floor contractions through visual, auditory, or tactile cues) (Fitz et al., 2012). Although the combination of ES, BF, and PFMT has been increasingly applied in clinical practice, high-quality meta-analyses remain lacking to systematically evaluate whether this combined intervention provides superior effectiveness compared with PFMT alone across several key multidimensional outcomes: PFMS (assessed by General Report Revised Urinary Grade (GRRUG) (Rao et al., 2023), graded 0–5), SUI incidence, POP severity (assessed by the POP-Q staging system, grades 0–IV), and sexual function (evaluated using the Female Sexual Function Index, FSFI). The findings of existing primary studies are heterogeneous, and comprehensive comparative analyses of these core outcomes are still limited.

## 2 Materials and methods

### 2.1 Registration

This systematic review and meta-analysis was conducted in accordance with the *Preferred Reporting Items for Systematic Reviews and Meta-Analyses (PRISMA) 2020* guidelines and was registered in the International Prospective Register of Systematic Reviews (PROSPERO) on August 27, 2025 (registration number: CRD420251134152). The completed PRISMA 2020 checklist is provided in **Supplementary Table 1**.

### 2.2 Ethical considerations

This study was conducted using data extracted from previously published studies. No new human or animal participants were recruited or examined. Therefore, ethical approval and informed consent were not required.

### 2.3 Inclusion and exclusion criteria

This review adopted the PICOS (Participants, Interventions, Comparisons, Outcomes, and Study design) framework to define eligibility criteria. In brief, studies were included if they enrolled postpartum women with PFD-related symptoms, used ES+BF combined with PFMT as the intervention, used PFMT alone as the comparator, reported at least one prespecified outcome, and adopted an RCT design. Studies were excluded if they were duplicate publications, lacked extractable outcome data, did not meet the predefined PICOS criteria, did not actually apply the combined ES+BF+PFMT intervention, or reported none of the three core treatment-schedule descriptors—intervention duration, treatment frequency, and session time. Baseline characteristics and baseline outcome values were reviewed descriptively to assess group comparability, but baseline statistical significance was not used as a mandatory exclusion criterion.

#### 2.3.1 Participants

Eligible participants were women within 12 months postpartum, regardless of parity (primipara or multipara) or mode of delivery (cesarean or vaginal). The 12-month postpartum window was selected because the first postpartum year is a clinically relevant period for pelvic floor recovery and rehabilitation, during which pelvic floor muscle recovery and pelvic organ support changes are commonly evaluated (Chen et al., 2013; Bø et al., 2022). Parity and mode of delivery were not used as eligibility restrictions because this review focused on the overall effect of ES+BF+PFMT in postpartum women with PFD-related symptoms rather than parity- or delivery mode-specific effects. Women presenting with one or more symptoms of pelvic floor muscle weakness, stress urinary incontinence, pelvic organ prolapse, or sexual dysfunction were considered eligible, as these symptoms correspond directly to the four primary outcomes of this study.

#### 2.3.2 Interventions

Included studies were required to explicitly describe the experimental intervention as ES and BF combined with PFMT. The comparison of interest was the additional effect of ES and BF when added to PFMT, rather than ES or BF alone. Therefore, studies were eligible only when PFMT was included in the experimental group and PFMT alone was used in the control group. Studies that used the term “ES+BF” in titles or abstracts but did not actually apply both ES and BF, or did not include PFMT as part of the experimental intervention, were excluded. When available, intervention duration, treatment frequency, and single-session treatment time were extracted and summarized in **Table 1**. If one or more of these details were not reported, the corresponding items were recorded as “not reported,” and partial missingness alone was not an exclusion criterion when the intervention components were clearly identifiable. Studies reporting none of the three core treatment-schedule descriptors (duration, frequency, and session time) were excluded.

**Table 1:** Basic characteristics of the included studies.

The first author/publication year	Country	Sample size (T/C)	Intevention(T/C)	ES+BF parameters			Follow-up time	Outcome
				Duration	Frequency	Session time		
(Zeng et al., 2022)	China	50/50	ES+BF+Kegel/Kegel	12 weeks	2×/week	Not reported	After 12-week treatment	①③
(Chu et al., 2012)	China	76/75	ES+BF+Kegel/Kegel	24 sessions	2-3×/week	20-30min	6 months postpartum	②③
(Gao et al., 2023)	China	68/67	ES+BF+ VC/VC	2 months	2×/week	Not reported	After 2-month treatment	③
(He et al., 2022)	China	100/100	ES+BF+ VC/VC	ES:5–7 weeks BF:10 weeks	ES:2×/week BF:1×/week	ES:30min BF:60min	After 10-week treatment	③
(Hu et al., 2022)	China	52/52	ES+BF+Kegel/Kegel	8 weeks	2×/week	30min	After 8-week treatment	①②③
(Huang et al., 2018)	China	62/61	ES+BF+Kegel/Kegel	6 weeks	2×/week	30min	18 weeks post-6-week treatment	①
(Jiang and Fu, 2018)	China	60/60	ES+BF+Kegel/Kegel	6 weeks	2×/week	30min	After 6-week treatment	③
(Li et al., 2015)	China	29/29	ES+BF+ VC/VC	3 months	Not reported	20min	After 3-month treatment	①
(Li et al., 2024)	China	327/343	ES+BF+Kegel/Kegel	Not reported	2-3×/week	Not reported	After treatment	③
(Liu et al., 2018)	China	100/100	ES+BF+Kegel/Kegel	8 weeks	2×/week	20-30min	After 8-week treatment	①
(Ruan et al., 2019)	China	80/80	ES+BF+Kegel/Kegel	6 months	2×/week	20-30min	After 6-month treatment	④
(Sun et al., 2017)	China	32/32	ES+BF+Kegel/Kegel	6 weeks	7×/week	50min	After 6-week treatment	①
(Zhang et al., 2020)	China	60/60	ES+BF+ VC/VC	8 weeks	2×/week	30min	After 8-week treatment	①③
(Tao et al., 2025)	China	36/34	ES+BF+ VC/VC	3 months	2×/week	25-30min	After 3-month treatment	②④
(Wang and Zhao, 2023)	China	24/24	ES+BF+Kegel/Kegel	12 weeks	2×/week	Not reported	After 12-week treatment	①
(Xia et al., 2019)	China	52/50	ES+BF+Kegel/Kegel	4 weeks	2×/week	15-30min	3 months postpartum	③
(Xu and Wei, 2019)	China	42/42	ES+BF+Kegel/Kegel	10–12 weeks	2×/week	30min	After treatment	④
(Yan et al., 2022)	China	40/40	ES+BF+Kegel/Kegel	8 weeks	2×/week	15-30min	After 8-week treatment	③
(Yang et al., 2024)	China	73/73	ES+BF+Kegel/Kegel	8 weeks	3×/week	35min	After 8-week treatment	④
(Zhang et al., 2012)	China	60/60	ES+BF+Kegel/Kegel	6 weeks	2×/week	20-30min	12 months postpartum	①②
(Zhang et al., 2020)	China	49/49	ES+BF+Kegel/Kegel	8 weeks	2×/week	15-20min	After 8-week treatment	④
(Zhao et al., 2019)	China	90/58	ES+BF+Kegel/Kegel	12 weeks	2×/week	30min	3 months postpartum	③
(Zhao et al., 2024)	China	45/45	ES+BF+Kegel/Kegel	8 weeks	3×/week	20min	After 8-week treatment	②
(Zhu et al., 2020)	China	80/80	ES+BF+Kegel/Kegel	10–15 sessions	2×/week	30min	After treatment	②③
(Ding et al., 2022)	China	34/32	ES+BF+Kegel/Kegel	10 sessions	1×/2 days	30min	8 weeks from baseline	②
(Meng et al., 2025)	China	39/39	ES+BF+Kegel/Kegel	2 months	2×/week	BES:10–15min; BF:10–20min	After 2-month treatment	④
(Li et al., 2025a)	China	75/75	ES+BF+Kegel/Kegel	3 months	2×/week	30min	After 3-month treatment	①
(Chen et al., 2025)	China	54/54	ES+BF+Kegel/Kegel	10 sessions	2×/week	20min	After 5-week treatment	④
(Cai, 2025)	China	60/60	ES+BF+Kegel/Kegel	12 weeks	2-3×/week	20-30min	After 12-week treatment	②④

T, treatment group; C, control group; ES, electrical stimulation; BF:biofeedback; VC, vaginal cone; ①, GRRUG perineal muscle strength test (0–5 grade); ②, Stress urinary incontinence incidence; ③, POP-Q stage (0–4 grade); ④, FSFI score.

#### 2.3.3 Comparators

Control groups were required to receive PFMT alone, without ES or BF. PFMT mainly included traditional Kegel exercises or vaginal cone training. Vaginal cone training was regarded as a resistance-based form of PFMT, aiming to enhance the training effect through added resistance.

#### 2.3.4 Outcomes

The following four core outcomes were prespecified: PFMS: a core pathological indicator, assessed using the GRRUG perineal muscle strength test (grades 0–5), directly reflecting pelvic floor muscle function; Stress urinary incontinence (SUI) incidence: a key symptomatic indicator, defined as the proportion of participants diagnosed with SUI at the post-intervention or follow-up assessment; it was not interpreted as strictly new-onset SUI because baseline SUI status, persistence, remission, and new-onset cases were not consistently reported; Pelvic organ prolapse (POP) severity: a structural abnormality indicator, assessed using the POP-Q staging system (stages 0–IV), providing objective quantification of organ position; Sexual function: a patient-reported outcome, assessed using the Female Sexual Function Index (FSFI), directly reflecting satisfaction with sexual life.To ensure comparability of results and minimize measurement bias, standardized assessment tools for each outcome were strictly required. To ensure meta-analysis feasibility, we extracted the outcome data as reported by the primary studies. It is important to acknowledge that for pelvic floor muscle strength, all studies used the GRRUG scale. We note that this scale is not internationally standardized like the Modified Oxford Scale (MOS) recommended by the International Continence Society. Furthermore, detailed parameters of the interventions (e.g., ES waveform, frequency, pulse width, current amplitude, electrode type and placement; BF modality [EMG vs. pressure]; supervision level of PFMT) were often inconsistently or incompletely reported across studies. This incomplete reporting prevented the planned subgroup analyses based on these technical parameters and constitutes a limitation of this review.

#### 2.3.5 Study design

Only randomized controlled trials (RCTs) were eligible.

#### 2.3.6 Exclusion criteria

We excluded duplicate publications, studies without extractable outcome data, studies that did not meet the predefined PICOS criteria, and studies in which the intervention and comparator could not be clearly identified as ES+BF+PFMT versus PFMT alone. Baseline characteristics and baseline outcome values were assessed descriptively to evaluate group comparability, but baseline statistical significance was not used as a mandatory exclusion criterion. No language restrictions were applied. Studies that reported none of the intervention duration, frequency, or session time were also excluded.

### 2.4 Literature search strategy

A comprehensive electronic search was conducted in PubMed, Web of Science, Embase, Cochrane Library, SinoMed, CNKI, WanFang, and VIP databases up to July 9, 2026. Both subject headings and free-text terms were applied. Considering the substantial heterogeneity in terminology describing the combined intervention of “electrical stimulation + biofeedback + pelvic floor muscle training” across publications, a two-step strategy was used for the English-language databases to ensure sensitivity and minimize missed studies: Core search terms: Free-text and subject-heading combinations were developed targeting the key intervention elements (“electrical stimulation,” “biofeedback”) and the target population (“postpartum”);Manual verification: Full texts of preliminarily identified studies were manually screened to confirm explicit inclusion of “pelvic floor muscle training” in the intervention protocol. For the Chinese-language databases, PFMT-related terms were included directly in the search strategy to improve search precision.

Representative English search terms included “biofeedback,” “biological feedback,” “physiological feedback,” “feedback,” “electric stimulation,” “electrical stimulation,” “postpartum period,” and “postpartum.” Detailed search strategies for each database are provided in the **Supplementary Table 2**. Search results from all databases were imported into EndNote (Version 21) for deduplication. After removing duplicates, two reviewers independently screened the retrieved studies according to the predefined inclusion and exclusion criteria.

### 2.5 Data extraction

Data from the included studies were independently extracted and summarized by two reviewers in a double-blind manner using a standardized data extraction form. Any discrepancies were resolved through discussion with a third reviewer. The extracted information included: first author, year of publication, country, sample size (intervention/control groups), intervention details, intervention duration, comparator, follow-up period, and outcome measures. Follow-up time points were extracted as reported in the original trials and summarized in **Table 1**.

### 2.6 Statistical analysis

All statistical analyses were performed using Review Manager 5.4 and Stata 16. Pooled effect sizes, subgroup analyses, sensitivity analyses, and publication bias assessments and certainty-of-evidence assessment were conducted. Post-intervention or follow-up outcome data were synthesized as reported in the original trials. Baseline characteristics and baseline outcome values were reviewed descriptively to assess group comparability, but baseline P values were not used to determine study eligibility. Because follow-up time points were variably reported across studies and the number of studies within each outcome was limited, formal short-, intermediate-, and long-term subgroup analyses were not performed. Differences in follow-up timing were therefore considered when interpreting heterogeneity and study limitations. For outcomes summarized as scores or scale values, including PFMS, POP severity, and sexual function, mean difference (MD) with 95% confidence intervals (CIs) was calculated. GRRUG pelvic floor muscle strength is an ordinal but semi-quantitative score, with increasing grades reflecting progressively greater muscle contraction, longer contraction duration, more repeated contractions, and greater resistance. In addition, several GRRUG studies reported only mean values and standard deviations rather than complete grade distributions, which limited the feasibility of a categorical synthesis across all included GRRUG studies. Therefore, the GRRUG outcome was synthesized using MD and interpreted as the average difference in pelvic floor muscle strength score between groups. POP-Q stage is also ordinal, and its stages should not be interpreted as strictly equally spaced clinical categories. Therefore, POP-Q was analyzed using two complementary approaches. First, an average-stage analysis was performed using MD to compare the overall difference in mean POP-Q stage score between groups across the full 0–IV stage distribution. This analysis was used to evaluate the general direction of shift in POP-Q severity, rather than to represent an exact transition between clinical stages. Second, a categorical sensitivity analysis was conducted to improve clinical interpretability. POP-Q stage was dichotomized as stage 0–I versus stage II–IV, and residual POP-Q stage II or higher at post-intervention or follow-up assessment was treated as the event. Risk ratios (RRs) with 95% CIs were pooled for this categorical analysis. For dichotomous outcomes—SUI incidence—risk ratio (RR) with 95% CI was used.

Statistical significance of pooled estimates was assessed using P values. Heterogeneity was quantified using the I^2^ statistic: ${\text{I}}^{2}\leq \;50\%$ was considered low, $\;50<{\text{I}}^{2}<75\%$ moderate, and ${\text{I}}^{2}\geq 75\%$ high heterogeneity. Model selection was based on statistical heterogeneity as well as clinical and methodological considerations. Tests for subgroup differences were performed for PFMS subgroup analyses, and subgroup findings were interpreted cautiously when the number of studies within subgroups was limited. When heterogeneity was substantial, subgroup and sensitivity analyses were performed to explore potential sources.

For outcomes with at least 10 included studies, publication bias was evaluated using funnel plots combined with Begg’s test and Egger’s test. When funnel plot asymmetry or statistically significant small-study effects were detected, the trim-and-fill method was further applied. The certainty of evidence for each main outcome was assessed using the Grading of Recommendations Assessment, Development and Evaluation (GRADE) approach and categorized as high, moderate, low, or very low.

## 3 Results

### 3.1 Study selection results

**Figure 1:**
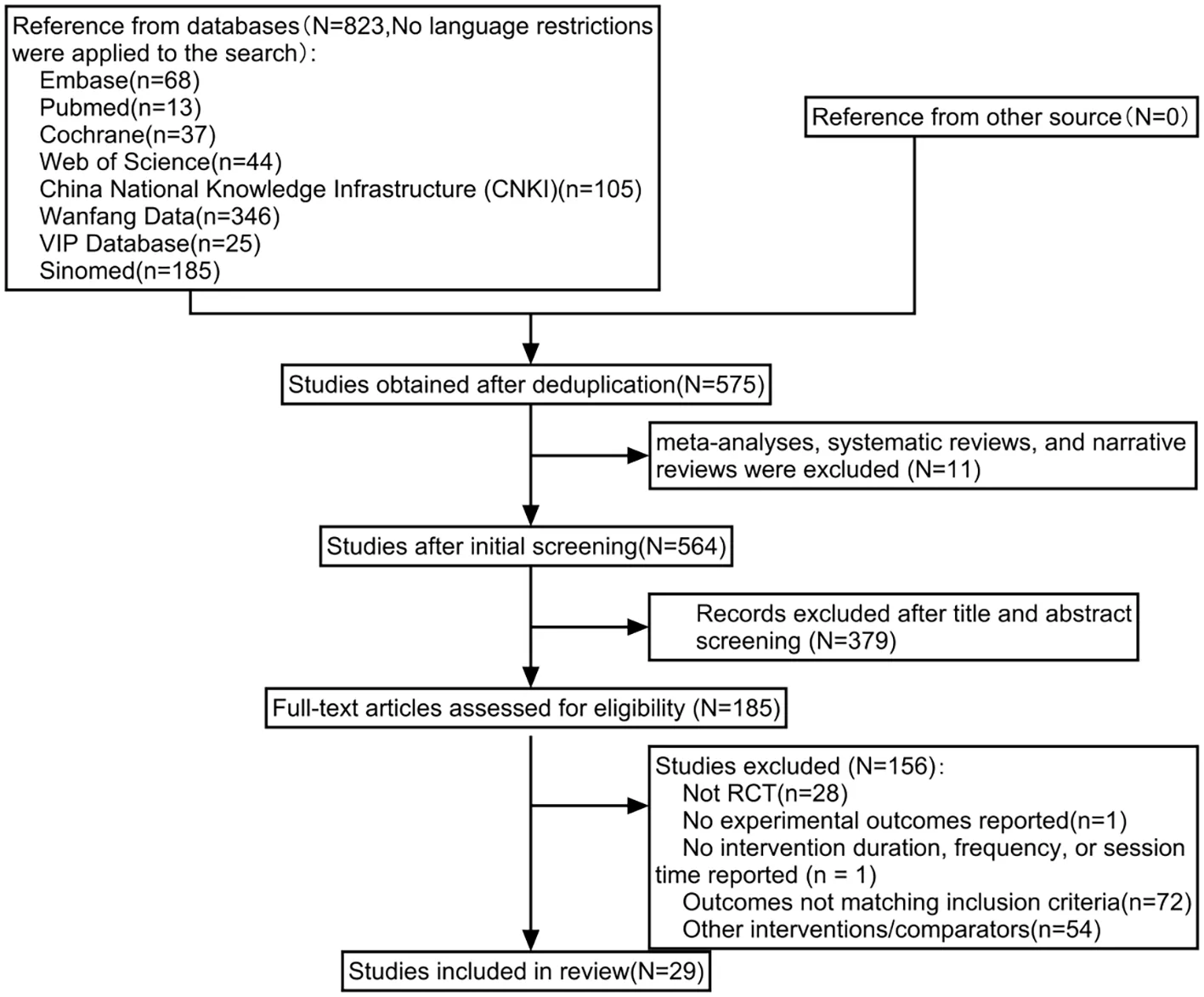
Flowchart of the literature screening process.

A total of 823 records were identified: 68 from Embase, 13 from PubMed, 37 from Cochrane Library, 44 from Web of Science, 105 from CNKI, 346 from WanFang, 25 from VIP, and 185 from SinoMed. No additional records were retrieved from other sources. After removing duplicates, 575 studies remained. Subsequently, 11 meta-analyses, systematic reviews, or narrative reviews were excluded, leaving 564 records for title and abstract screening. Of these, 379 were excluded as irrelevant, and 185 full-text articles were assessed for eligibility. After full-text review, 156 articles were excluded for the following reasons: non-randomized controlled trials (n=28), absence of reported results (n=1), no intervention duration, frequency, or session time reported (n = 1), outcomes not meeting eligibility criteria (n=72), or interventions/comparators inconsistent with inclusion criteria (n=54).Finally, 29 randomized controlled trials (RCTs) were included in the analysis. The study selection process is illustrated in **Figure 1**.

### 3.2 Basic characteristics of the included studies

A total of 29 studies were ultimately included in the analysis. It is a critical characteristic of the evidence base that all included studies were conducted in China. This limits the generalizability of the findings to other populations and healthcare settings with different cultural, clinical, and device-related practices in postpartum care. Furthermore, the relatively short total intervention durations (as brief as 4 weeks in some studies, see **Table 1**) suggest that intravaginal electrical stimulation may have been initiated early in the postpartum period, a practice that warrants caution given international guideline recommendations. Among them, 10 studies reported PFMS assessed by the GRRUG perineal muscle strength test, 8 studies reported stress urinary incontinence (SUI) incidence diagnosed according to International Continence Society standards or the ICI-Q-SF questionnaire, 12 studies reported pelvic organ prolapse (POP) severity using the POP-Q staging system, and 8 studies reported sexual function outcomes measured by the FSFI. The baseline characteristics of the included studies are summarized in **Table 1**.

### 3.3 Risk-of-bias and methodological quality assessment

The risk of bias of the 29 included studies was evaluated using the Cochrane domain-based risk-of-bias tool, and an overall methodological-quality category was assigned according to previously published rating standards. Two reviewers independently assessed the risk of bias for each included study. Disagreements were resolved through discussion or by consultation with a third reviewer. Nine studies did not report randomization methods. Among the remaining 20, one study employed a lottery randomization method, two used simple randomization, and 17 adopted a random number table method. None of the studies reported allocation concealment or blinding procedures. Regarding data completeness, four studies reported attrition rates; two specified similar reasons for loss to follow-up, while the other two failed to provide detailed reasons or related information.

Following previously published rating standards (Xin et al., 2026), the descriptive overall methodological-quality category was classified as follows: High quality: no items rated as high risk, and ≤3 items rated as “unclear risk”; Moderate quality: only one item rated as high risk with no additional unclear-risk items, or no high-risk items but ≥4 items rated as “unclear risk”; Low quality: all remaining cases, including studies with one high-risk item combined with one or more unclear-risk items.

**Figure 2:**
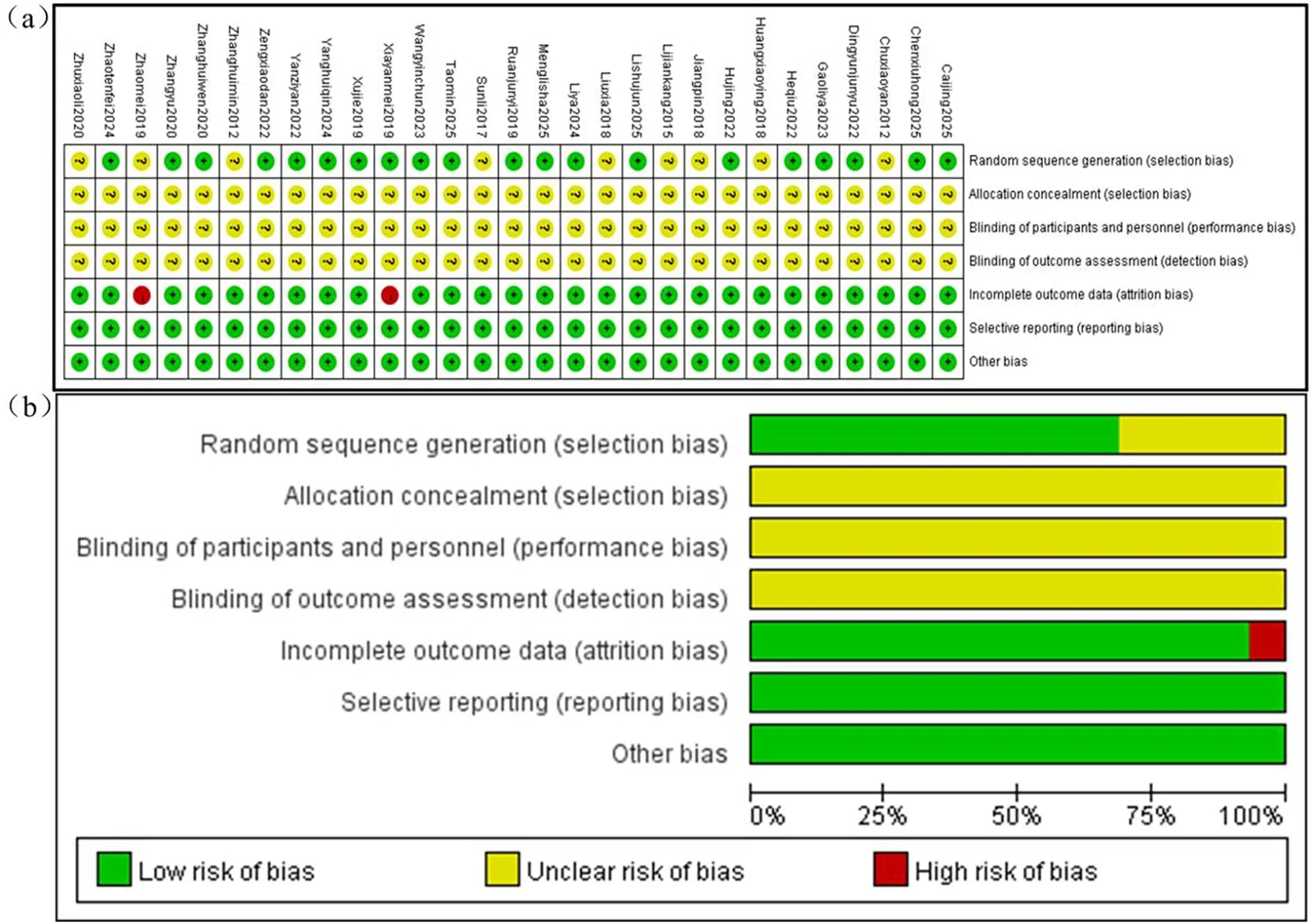
**(a)** Risk of bias summary. **(b)** Risk of bias graph.

Based on this framework, 19 studies were classified into the high methodological-quality category, 8 into the moderate category, and 2 into the low category. This overall methodological-quality category was descriptive and should not be interpreted as indicating low risk of bias across all individual domains. Because allocation concealment and blinding procedures were not reported in any included study, these methodological limitations were further considered in the GRADE assessment of the certainty of evidence. The results are illustrated in **Figure 2**.

### 3.4 Meta-analysis results

#### 3.4.1 PFMS

Ten studies (n = 1087 postpartum women) were included in the analysis of PFMS. The pooled results showed that the combined intervention (ES + BF + PFMT) was significantly more effective than PFMT alone in improving PFMS as measured by the GRRUG score (MD = 0.85, 95% CI: 0.69–1.01, p < 0.00001), although moderate heterogeneity was observed (I^2^ = 64%). Because the GRRUG scale is a semi-quantitative ordinal score and several included studies reported only mean values and standard deviations, the pooled effect was interpreted as an average difference in pelvic floor muscle strength score rather than a precise shift between muscle-strength grades.

To explore the source of heterogeneity, subgroup analyses were performed. When stratified by training modality, the vaginal cone subgroup (2 studies) showed a pooled MD of 0.95 (95% CI: 0.61–1.30; I^2^ = 36%), whereas the Kegel subgroup (8 studies) showed a pooled MD of 0.83 (95% CI: 0.65–1.01; I^2^ = 67%). The test for subgroup differences showed no significant difference between training modalities (Chi^2^ = 0.40, df = 1, P = 0.53).

Further subgroup analyses based on training duration within the Kegel subgroup revealed: 6-week programs (3 studies): MD = 0.91 [0.65, 1.17], I^2^ = 71%;8-week programs (2 studies): MD = 0.54 [0.35, 0.72], I^2^ = 0%;12-week programs (3 studies): MD = 0.94 [0.68, 1.19], I^2^ = 16%.Sensitivity analysis indicated that one study (Zhanghuimin, 2012), with a 12-month postpartum follow-up period, differed substantially from the other two 6-week trials that assessed outcomes immediately post-intervention. This study appeared to be an important contributor to heterogeneity. After its exclusion, the pooled effect for the 6-week group was MD = 0.75 [0.59, 0.92], with heterogeneity reduced to I^2^ = 0%.

Notably, the effect size for the 6-week group remained higher than that of the 8-week group (0.75 vs. 0.54), suggesting that factors such as training intensity, baseline PFMS, and participant characteristics may play a more critical role than duration alone. The test for subgroup differences across Kegel training duration subgroups was significant (Chi^2^ = 8.46, df = 2, P = 0.01). The 6-week and 12-week Kegel subgroups showed larger pooled effects than the 8-week subgroup. However, because the number of studies within each subgroup was limited, these subgroup findings should be interpreted cautiously rather than as confirmatory evidence of superiority.

**Figure 3:**
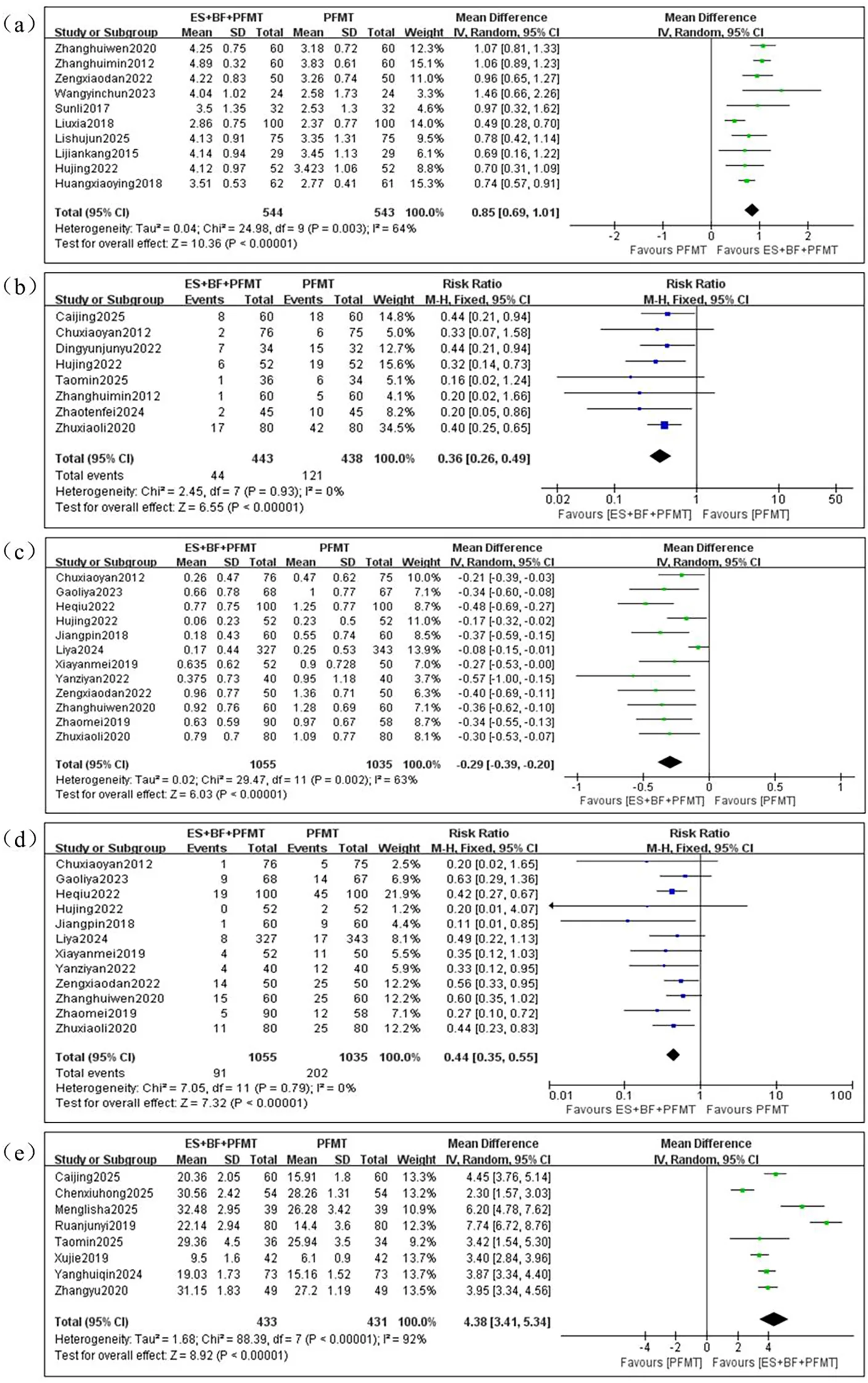
Forest plots of the main outcomes and POP-Q categorical sensitivity analysis. **(a)** Pelvic floor muscle strength assessed by GRRUG score; **(b)** SUI incidence at post-intervention or follow-up assessment; **(c)** POP-Q average-stage analysis using mean difference; **(d)** categorical sensitivity analysis of POP-Q stage, with residual POP-Q stage II or higher treated as the event; **(e)** sexual quality of life assessed by FSFI score.

The pooled results are presented in **Figure 3a**, and subgroup as well as sensitivity analysis outcomes are summarized in **Table 2**.

**Table 2:** Subgroup and sensitivity analysis result of PFMS.

Category	Subgroup	Number of studies (n)	Effect size (MD)	95%CI	I^2^ (%)	p-value
By intervention type	Vaginal cone	2	0.95	0.61-1.30	36	P<0.00001
	Kegel	8	0.83	0.65-1.01	67	P<0.00001
By training duration (Kegel)	6 weeks	3	0.91	0.65-1.17	71	P<0.00001
	8 weeks	2	0.54	0.35-0.72	0	P<0.00001
	12 weeks	3	0.94	0.68-1.19	16	P<0.00001
Sensitivity analysis (6-week Kegel)	excluding Zhanghuimin, 2012	2	0.75	0.59-0.92	0	P<0.00001

Tests for subgroup differences showed no significant difference between training modalities (Chi^2^ = 0.40, df = 1, P = 0.53), but a significant difference across Kegel training duration subgroups (Chi^2^ = 8.46, df = 2, P = 0.01).

#### 3.4.2 SUI incidence

Eight studies (n = 881 postpartum women) evaluated the effect of electrical stimulation combined with biofeedback and pelvic floor muscle training (ES + BF + PFMT) compared with PFMT alone on the incidence of stress urinary incontinence (SUI). The pooled analysis demonstrated that the combined intervention significantly reduced the proportion of participants with SUI (RR = 0.36, 95% CI: 0.26–0.49, Z = 6.55, p < 0.00001). Heterogeneity was negligible (I^2^ = 0%), suggesting good consistency among the included studies.

The forest plot of the combined results is shown in **Figure 3b**.

#### 3.4.3 POP severity

**Figure 4:**
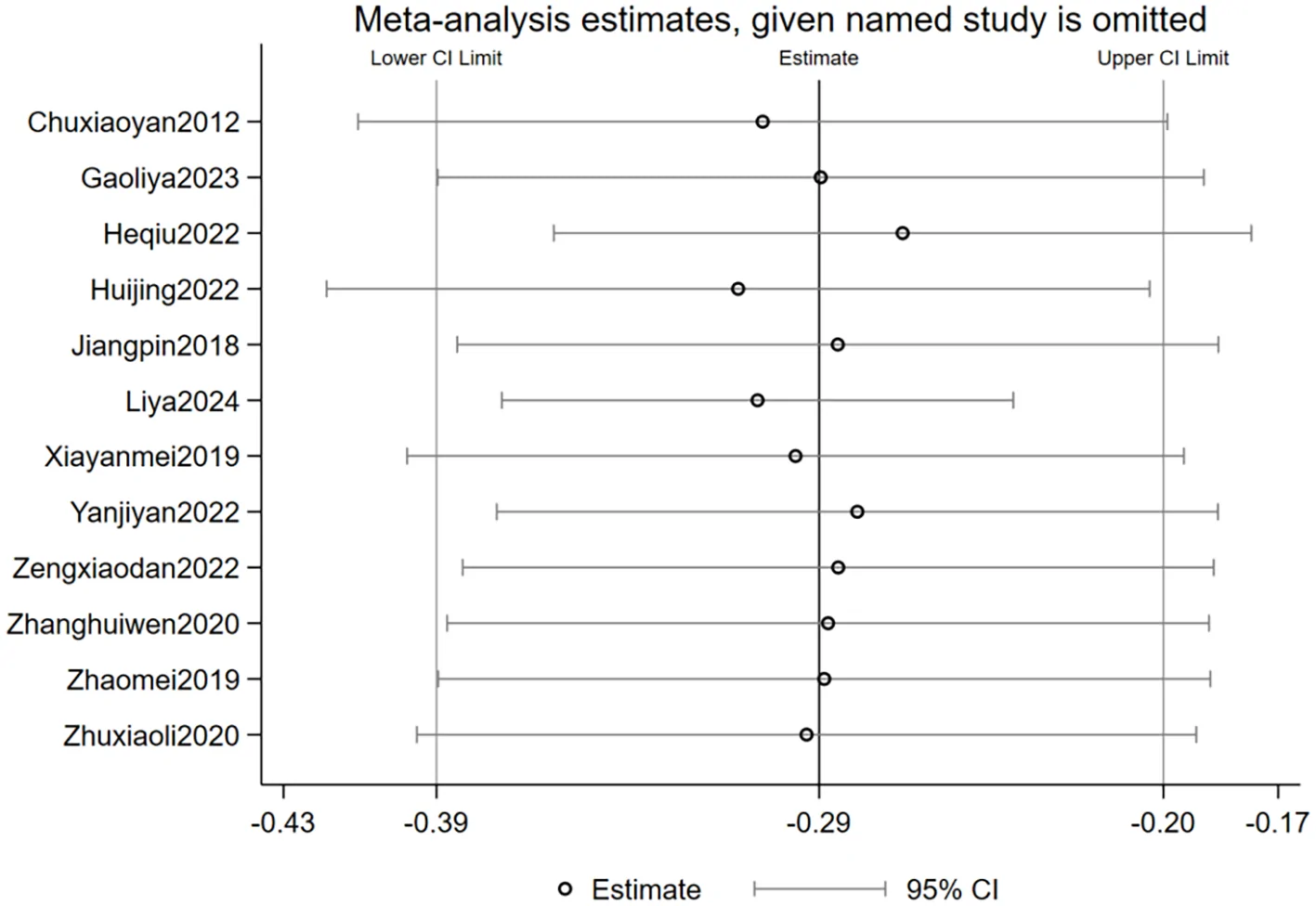
Sensitivity analysis of POP.

Twelve studies (n = 2090 postpartum women) were included in the analysis of pelvic organ prolapse (POP). The pooled results indicated that the combined intervention (ES + BF + PFMT) significantly reduced POP-Q stage compared with PFMT alone (MD = −0.29, 95% CI: −0.39 to −0.20, Z = 6.03, p < 0.00001). While statistically significant, the clinical interpretability of a POP-Q stage reduction of 0.2-0.3 is uncertain in the absence of a pre-specified minimal clinically important difference (MCID) for this population. The clinical relevance of this magnitude of change should be interpreted with caution. However, moderate heterogeneity was observed (I^2^ = 63%), the pooled results are presented in **Figure 3c**. To identify sources of heterogeneity, sensitivity analyses were performed (see **Figure 4**). Although the study by Liya (2024) did not appear as an obvious outlier in the sensitivity plot, its effect size (MD = −0.08) was notably lower than those of other studies. Moreover, it only reported training frequency (twice per week) without specifying total intervention duration or single-session length, making the protocol difficult to compare with other trials.

After excluding this study, the pooled effect size was MD = −0.31 (95% CI: −0.37 to −0.24, p < 0.00001), and heterogeneity decreased to I^2^ = 0%, suggesting that the Liya (2024) trial may have been an important contributor to heterogeneity. Sensitivity analysis outcomes are summarized in **Table 3**.

**Table 3:** Sensitivity analyses result of pelvic organ prolapse.

Analysis	Studies (n)	Participants (ES+BF+PFMT/PFMT)	Effect size (MD)	95%CI	I^2^ (%)		p-value
Overall analysis (all included studies)	12	1055/1035	-0.29	[-0.39, -0.20]	63	P<0.00001	
After excluding Liya (2024)	11	728/692	-0.31	[-0.37, -0.24]	0	P<0.00001	

Because POP-Q stage is ordinal, POP-Q was further examined using a categorical sensitivity analysis to improve clinical interpretability. Residual POP-Q stage II or higher at post-intervention or follow-up assessment was treated as the event. The categorical analysis showed a direction of effect consistent with the average-stage MD analysis. The proportion of residual stage II or higher POP-Q was lower in the ES+BF+PFMT group than in the PFMT-alone group (91/1055 vs. 202/1035; RR = 0.44, 95% CI: 0.35 to 0.55, P < 0.00001; I^2^ = 0%; **Figure 3d**). Therefore, the categorical sensitivity analysis supported the direction of the average-stage analysis. However, the POP-Q findings should still be interpreted cautiously because POP-Q stage is ordinal, the clinical meaning of small average stage-score differences is uncertain, and the certainty of evidence for this outcome was very low.

#### 3.4.4 Sexual quality of life

Eight studies (n = 864 postpartum women) assessed the effect of the combined intervention on sexual quality of life. The pooled analysis showed that ES + BF + PFMT was significantly more effective than PFMT alone (MD = 4.38, 95% CI: 3.41–5.34, p < 0.00001). However, heterogeneity across studies was substantial (I^2^ = 92%). The pooled results are illustrated in **Figure 3e**.

**Figure 5:**
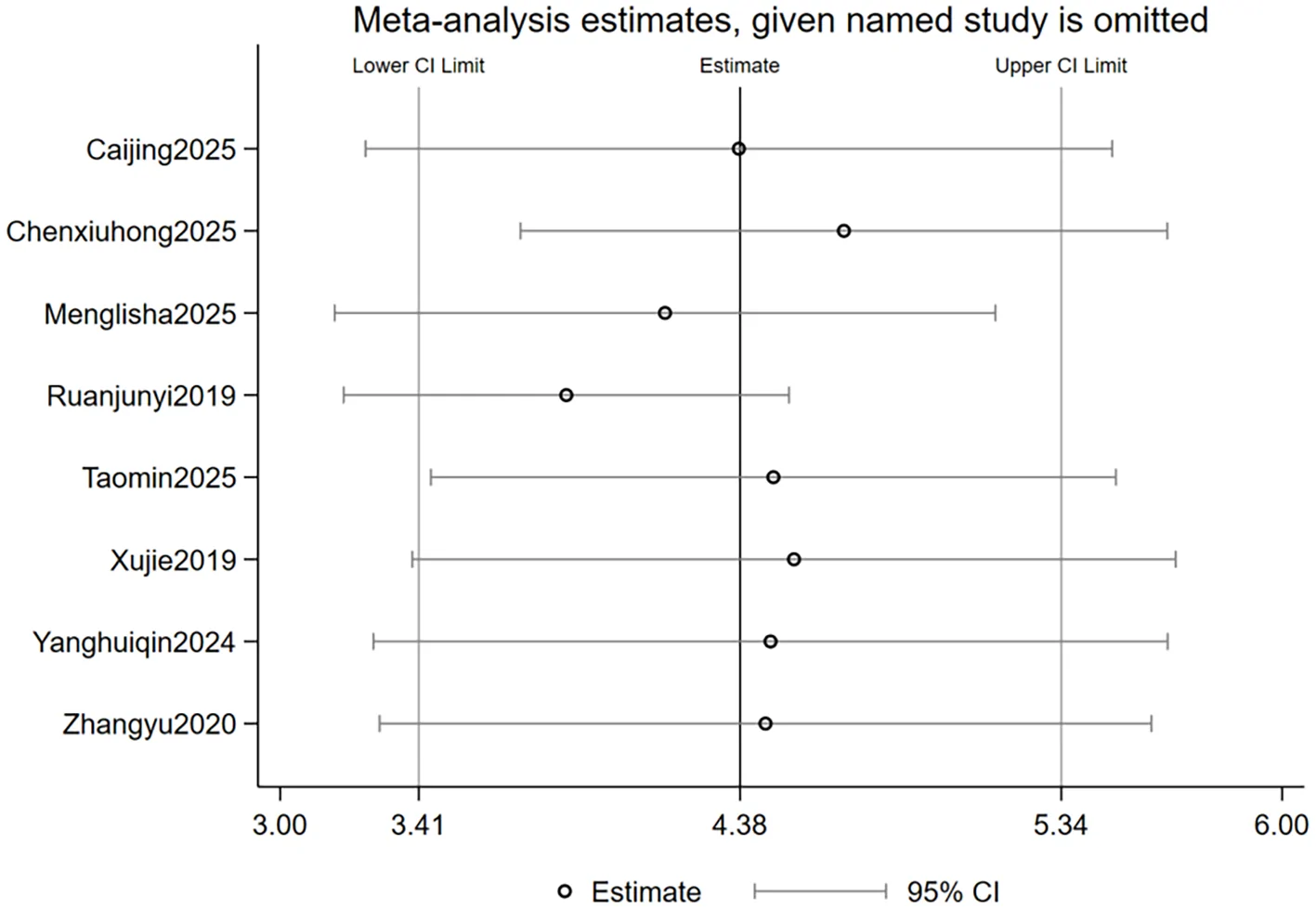
Sensitivity analysis of sexual quality of life.

Leave-one-out sensitivity analysis was performed to examine the influence of individual studies (**Figure 5**). After omitting each study in turn, the pooled effects remained in favor of ES + BF + PFMT and remained statistically significant, with pooled MDs ranging from 3.86 to 4.69 (**Table 4**). However, heterogeneity remained substantial across all leave-one-out analyses, with I^2^ values ranging from 82% to 93%. These findings indicated that the high heterogeneity in FSFI was not driven by any single study, although the direction and statistical significance of the pooled effect were stable. Therefore, all eight eligible studies were retained in the primary analysis. Potential clinical and methodological sources of heterogeneity are further discussed in the Discussion section.

**Table 4:** Sensitivity analysis result of sexual quality of life.

Analysis	Studies (n)	Participants (ES+BF+PFMT/PFMT)	Effect size (MD)	95%CI	I^2^ (%)	p-value
Overall analysis (all studies)	8	433/431	4.38	[3.41, 5.34]	92	P<0.00001
After excluding Caijing (2025)	7	373/371	4.37	[3.26, 5.49]	93	P<0.00001
After excluding Chenxiuhong (2025)	7	379/377	4.69	[3.72, 5.66]	91	P<0.00001
After excluding Menglisha (2025)	7	394/392	4.15	[3.16, 5.14]	92	P<0.00001
After excluding Ruanjunyi (2019)	7	353/351	3.86	[3.19, 4.52]	82	P<0.00001
After excluding Taomin (2025)	7	397/397	4.48	[3.45, 5.50]	93	P<0.00001
After excluding Xujie (2019)	7	391/389	4.54	[3.40, 5.68]	93	P<0.00001
After excluding Yanghuiqin (2024)	7	360/358	4.47	[3.28, 5.66]	93	P<0.00001
After excluding Zhangyu (2020)	7	384/382	4.45	[3.30, 5.61]	93	P<0.00001

The results of sensitivity analyses are summarized in **Table 4**.

#### 3.4.5 Publication bias assessment

Publication bias may inflate pooled effect estimates and affect the reliability of meta-analysis conclusions. In the revised analysis, funnel plots, Egger’s regression test, and Begg’s rank correlation test were applied only to outcomes with at least 10 included studies, in order to avoid unreliable interpretation when the number of studies was small. Therefore, formal publication bias assessment was performed for PFMS and POP-Q, but not for SUI incidence or sexual quality of life because fewer than 10 studies were available for these two outcomes.

**Figure 6:**
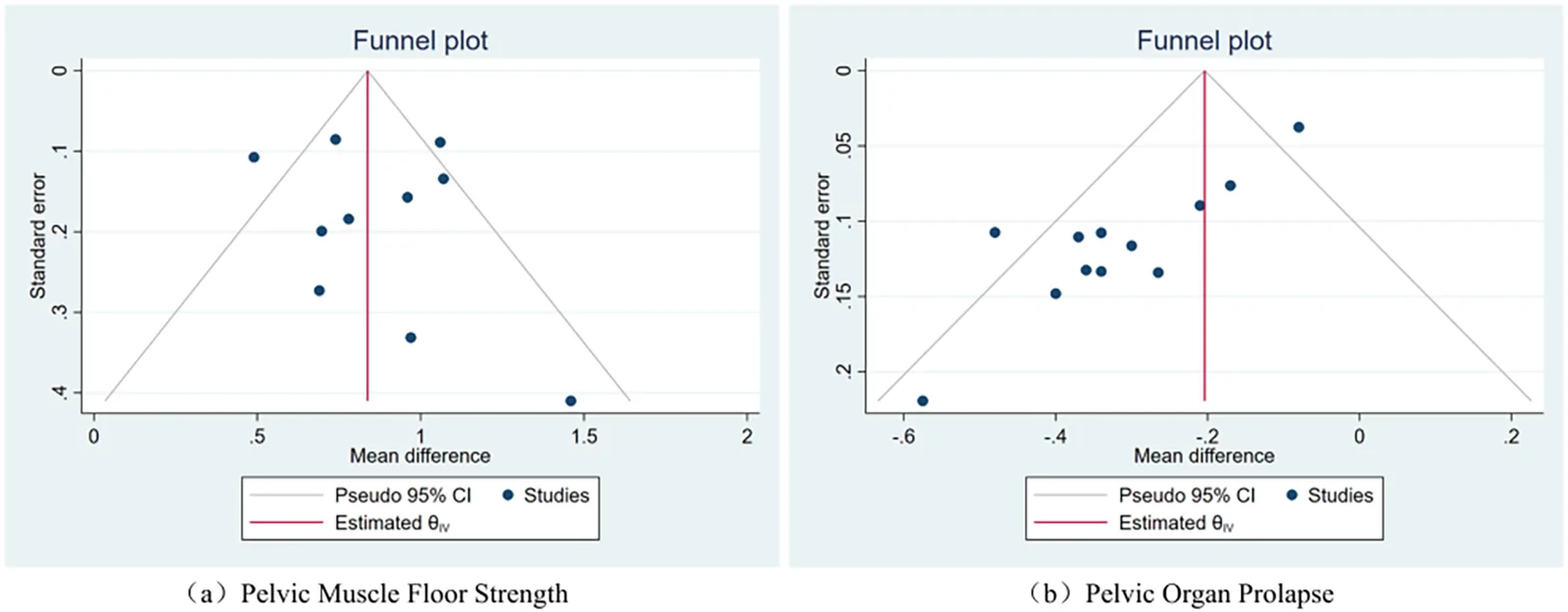
Funnel plots for publication bias assessment. **(A)** Pelvic floor muscle strength; **(B)** Pelvic organ prolapse.

**Figure 6** presents the funnel plots for PFMS and POP-Q. For PFMS, visual inspection showed a generally symmetrical distribution, suggesting no obvious publication bias. Egger’s test (P = 0.4399) and Begg’s test (P = 0.8580) were both nonsignificant, further indicating no statistically significant evidence of publication bias. Given these consistent results, the trim-and-fill method was not applied for PFMS. For POP-Q, visual inspection of the funnel plot suggested possible asymmetry. Egger’s test indicated potential publication bias (P < 0.0001), whereas Begg’s test did not reach statistical significance (P = 0.0865). Trim-and-fill analysis estimated six potentially missing studies. After adjustment, the pooled effect size changed from MD = −0.286 (95% CI: −0.372 to −0.199) to MD = −0.195 (95% CI: −0.282 to −0.109). This suggested that publication bias may have influenced the magnitude of the pooled effect, but the adjusted result remained statistically significant. For SUI incidence and sexual quality of life, formal funnel plot asymmetry tests were not performed because the number of included studies was fewer than 10. Therefore, publication bias for these two outcomes could not be reliably assessed. In summary, no obvious publication bias was detected for PFMS, whereas POP-Q showed evidence of possible publication bias. Publication bias could not be reliably evaluated for SUI incidence and sexual quality of life due to the limited number of included studies.

#### 3.4.6 Certainty of evidence

The certainty of evidence for each outcome was assessed using the GRADE approach and is summarized in **Table 5**. The certainty of evidence was moderate for SUI incidence, low for pelvic floor muscle strength and sexual quality of life, and very low for POP-Q stage.

**Table 5:** GRADE assessment of the certainty of evidence for the main outcomes.

Certainty assessment							№ of patients		Effect		Certainty	Importance
№ of studies	Study design	Risk of bias	Inconsistency	Indirectness	Imprecision	Other considerations	Electrical stimulation and biofeedback combined with pelvic floor muscle training	Pelvic floor muscle training alone	Relative (95% CI)	Absolute (95% CI)		
Pelvic floor muscle strength (assessed with: GRRUG perineal muscle strength test; Scale from: 0 to 5)												
10	randomised trials	serious	serious	not serious	not serious	none	544	543	–	MD 0.85 points higher (0.69 higher to 1.01 higher)	⨁⨁◯◯ Low	CRITICAL
SUI incidence (assessed with: Incidence of stress urinary incontinence)												
8	randomised trials	serious	not serious	not serious	not serious	none	44/443 (9.9%)	121/438 (27.6%)	RR 0.36 (0.26 to 0.49)	177 fewer per 1,000 (from 204 fewer to 141 fewer)	⨁⨁⨁◯ Moderate	CRITICAL
POP-Q stage (Scale from: 0 to 4)												
12	randomised trials	serious	serious	not serious	not serious	publication bias strongly suspected	1055	1035	–	MD 0.29 stages lower (0.39 lower to 0.20 lower)	⨁◯◯◯ Very low	CRITICAL
Sexual quality of life/FSFI (assessed with: FSFI score; Scale from: 2 to 36)												
8	randomised trials	serious	serious	not serious	not serious	none	433	431	–	MD 4.38 points higher (3.41 higher to 5.34 higher)	⨁⨁◯◯ Low	CRITICAL

CI, confidence interval; MD, mean difference; RR, risk ratio; PFMS, pelvic floor muscle strength; SUI, stress urinary incontinence; FSFI, Female Sexual Function Index. a. Downgraded by one level for risk of bias because allocation concealment and blinding were unclear in all included studies. b. Downgraded by one level for inconsistency because substantial heterogeneity was observed. c. Downgraded by one level for possible publication bias because Egger’s test indicated potential publication bias and trim-and-fill analysis estimated six potentially missing studies. d. Publication bias was not formally assessed for SUI incidence and sexual quality of life because fewer than 10 studies were available for these outcome.

## 4 Discussion

This meta-analysis systematically evaluated the therapeutic effects of ES and BF combined with PFMT in postpartum women with PFD. Four key outcomes—PFMS, SUI incidence, POP severity, and sexual quality of life—were compared with PFMT alone. These findings suggest that ES + BF + PFMT may provide multidimensional benefits in postpartum pelvic floor rehabilitation. However, GRADE assessment indicated moderate certainty for SUI incidence, low certainty for PFMS and sexual quality of life, and very low certainty for POP severity; therefore, findings should be interpreted with consideration of the certainty of evidence.

These findings are generally consistent with previous meta-analyses. Ji Jia (2020) (Ji et al., 2020) reported that combined interventions improved sexual function, SUI incidence, and POP severity compared with PFMT alone, while (Zhu et al., 2022) reported improved PFMS with ES + BF + PFMT. By incorporating additional eligible RCTs and using standardized outcome measures, including GRRUG strength testing, POP-Q staging, and FSFI scores, the present review updates and further supports the available evidence for key postpartum PFD outcomes.

The observed benefit of the combined intervention is biologically plausible. ES may facilitate passive pelvic floor muscle contractions and neuromuscular activation, particularly in women with poor voluntary contraction capacity, whereas BF may improve contraction awareness, technique, and adherence through real-time feedback (Wu et al., 2021; Li et al., 2025b). These modalities should be interpreted as adjuncts to, rather than replacements for, supervised PFMT, which remains the cornerstone and first-line conservative treatment for postpartum PFD. This interpretation is consistent with prior evidence showing that ES or BF alone may have limited or uncertain advantages over PFMT, supporting their role as complementary components within a combined rehabilitation strategy (Fitz et al., 2012; Chen et al., 2024). The timing of postpartum ES should also follow relevant safety recommendations.

Subgroup and sensitivity analyses suggested that intervention parameters may influence the magnitude of treatment effects. In the PFMS analysis, no significant subgroup difference was observed between vaginal cone and Kegel training, whereas Kegel training duration showed a significant subgroup difference. The 6-week and 12-week Kegel subgroups showed larger pooled effects than the 8-week subgroup. However, because the number of studies within each subgroup was limited, these findings should be interpreted cautiously. For the POP-Q outcome, heterogeneity remained moderate in the pooled analysis, indicating that differences in intervention reporting and patient characteristics should also be considered when interpreting structural outcomes. Future studies should standardize intervention protocols, clearly report ES/BF parameters and PFMT supervision, and employ stratified designs to ensure more precise effect estimates.

The FSFI analysis showed substantial heterogeneity (I^2^ = 92%). Leave-one-out sensitivity analysis indicated that, after omitting each study in turn, the pooled effects remained in favor of ES + BF + PFMT and remained statistically significant, with pooled MDs ranging from 3.86 to 4.69. However, heterogeneity remained substantial across all leave-one-out analyses (I^2^ range: 82%–93%), indicating that the high heterogeneity was not driven by any single study. This heterogeneity appeared to reflect differences in the magnitude rather than the direction of treatment effects, as all included studies favored ES + BF + PFMT. Several clinical and methodological factors may explain this heterogeneity, including differences in postpartum populations (PFD, SUI, or mild uterine prolapse), baseline FSFI scores, intervention duration (5 weeks to 6 months), ES/BF parameters, treatment frequency, and PFMT or rehabilitation protocols such as Kegel-based training, conventional pelvic floor rehabilitation, and vaginal cone training. Therefore, the high heterogeneity in FSFI was considered clinically plausible and was further reflected in the GRADE assessment as serious inconsistency.

Regarding publication bias, formal assessment was performed only for outcomes with at least 10 included studies. No obvious publication bias was detected for PFMS, as Egger’s test (P = 0.4399) and Begg’s test (P = 0.8580) were both nonsignificant. For POP-Q, funnel plot asymmetry and Egger’s test (P < 0.0001) suggested possible publication bias, whereas Begg’s test did not reach statistical significance (P = 0.0865). Trim-and-fill analysis estimated six potentially missing studies, and the adjusted pooled effect remained statistically significant. For SUI incidence and sexual quality of life, publication bias could not be reliably assessed because fewer than 10 studies were available for each outcome.

A strength of this review is its focus on a clearly defined conservative rehabilitation strategy, ES + BF combined with PFMT, across several clinically relevant postpartum PFD outcomes. Several limitations should also be considered. First, all 29 included RCTs were conducted in China, which limits generalizability to other populations and healthcare settings. Second, although an overall methodological-quality category was assigned according to a previously published standard, this category was descriptive and did not replace domain-level risk-of-bias judgments. Many studies insufficiently described randomization, and none reported allocation concealment or blinding; these limitations were considered in the GRADE assessment. Third, PFMS and POP severity were both assessed using ordinal scales, but their interpretability and available data formats differed. GRRUG pelvic floor muscle strength is a semi-quantitative ordinal score, with increasing grades reflecting progressively greater contraction duration, repetition, and resistance. However, several GRRUG studies reported only mean values and standard deviations rather than complete grade distributions, which limited the feasibility of a categorical synthesis across all GRRUG studies. In addition, the GRRUG scale lacks international validation compared with the ICS-recommended Modified Oxford Scale. In contrast, POP-Q stage is an anatomical ordinal classification. For POP-Q, the average-stage MD analysis was used to evaluate the overall direction of change in average stage score across the full 0–IV distribution, while a categorical sensitivity analysis was performed to improve clinical interpretability using residual stage II or higher as the event. This complementary approach supported the direction of the POP-Q finding but did not eliminate the limitations related to the ordinal nature of the outcome. Therefore, POP-Q results, particularly small average stage-score differences, should be interpreted cautiously. Fourth, incomplete reporting of ES/BF parameters and PFMT supervision limited planned subgroup analyses. Fifth, follow-up time points varied across studies, ranging from immediate post-treatment assessment to several months postpartum. Because the number of studies within each outcome was limited and follow-up definitions were inconsistent, time-specific subgroup analyses were not feasible. Therefore, the pooled estimates should be interpreted as overall post-intervention or follow-up effects rather than strictly short-, intermediate-, or long-term effects. Finally, substantial heterogeneity remained in some outcomes, particularly FSFI, and publication bias could not be reliably assessed for SUI incidence and sexual quality of life. Future studies should use standardized reporting, internationally recognized outcome measures, longer follow-up, and more diverse populations.

## 5 Conclusion

This systematic review and meta-analysis of 29 RCTs suggests that ES and BF combined with PFMT may provide additional benefits compared with PFMT alone for postpartum women with PFD-related symptoms. The most consistent evidence was observed for reducing SUI incidence, with moderate certainty of evidence. Potential benefits were also observed for PFMS and sexual quality of life, although the certainty of evidence was low and heterogeneity should be considered. The findings for POP-Q stage should be interpreted with particular caution because the certainty of evidence was very low and possible publication bias was detected.

Overall, ES and BF may be considered adjunctive modalities to PFMT rather than replacements for supervised PFMT. Future international, high-quality RCTs should use validated outcome measures, clearly report ES/BF/PFMT protocols, include longer and standardized follow-up, and follow safety guidance for the timing of postpartum electrical stimulation.

## Data Availability

The original contributions presented in the study are included in the article/**Supplementary Material**. Further inquiries can be directed to the corresponding author.
